# *Acanthamoeba polyphaga mimivirus* Virophage Seroconversion in Travelers Returning from Laos

**DOI:** 10.3201/eid1809.120099

**Published:** 2012-09

**Authors:** Philippe Parola, Aurélie Renvoisé, Elisabeth Botelho-Nevers, Bernard La Scola, Christelle Desnues, Didier Raoult

**Affiliations:** Author affiliation: Aix Marseille University, Marseille, France

**Keywords:** Mimivirus, giant virus, amoeba, ameba, virophage, viruses, Laos

## Abstract

During January 2010, a husband and wife returned from Laos to France with probable parasitic disease. Increased antibodies against an *Acanthamoeba polyphaga mimivirus* virophage indicated seroconversion. While in Laos, they had eaten raw fish, a potential source of the virophage. This virophage, associated with giant viruses suspected to cause pneumonia, could be an emerging pathogen.

An ameba-associated giant virus, *Acanthamoeba polyphaga mimivirus* (APM), was first described in 2003 after its discovery in water collected from a cooling tower in the United Kingdom (*1*). In 2008, a new APM strain (mamavirus), along with an APM virophage, was discovered in water from a cooling tower in France (*2*). The APM virophage is a virus that infects giant viruses. The pathogenicity of giant viruses is still a matter of debate (*3*). We describe seroconversion to antibodies against the APM virophage in 2 humans.

## The Study

The patients were a 29-year-old woman and her 36-year-old husband, each born in Laos, who had immigrated to France in 2008 and 2000, respectively. During December, 20, 2009, through January 22, 2010, they traveled to Laos with their 4-month-old baby to visit friends and relatives. This was their first return to Laos since immigration. While in Laos, they ate local food, including raw fish. Five days after their return to France, they experienced asthenia, low-grade fever, myalgia, and nausea. They had no other history of travel, and their baby showed no clinical signs.

Blood test results indicated hypereosinophilia, hepatic cytolysis, and cholestasis (Table). At 7 days after the onset of symptoms, the patients were hospitalized; physical examination of the man detected no abnormalities, and examination of the woman detected tachycardia. Echocardiography of the woman showed a thin pericardial effusion, but chest radiographs showed no abnormalities. Serum from the woman contained antibodies against toxocara, trichinellae, and *Fasciolia* trematodes; and serum from the man contained antibodies against *Fasciolia* trematodes. No parasites were recovered from stool samples from either patient. The patients received antiparasitic treatment (praziquantel for 3 days and albendazole for 15 days), after which they recovered completely and their eosinophil counts returned to reference levels.

**Table:** Biological parameters for 2 patients who seroconverted against *Acanthamoeba polyphaga mimivirus* virophage after visit to Laos, 2010*

Parameter	29-year-old woman								36-year-old man				
	Past samples	Outpatient visit	Day 1 hospitalization	Follow-up visits					Day 1 hospitalization	Follow-up visits			
	2009 Aug	2010 Jan 22	2010 Jan 29	2010 Feb 6	2010 Feb 23	2010 Mar 31	2010 May 3		2010 Jan 29	2010 Feb 4	2010 Feb 23	2010 Mar 31	2010 May 3
Leukocytes, × 10^9^/L	NT	14.32	37.9	16.7	7.2	NA	6.18		23.8	13.7	8.2	NA	7.6
Neutrophils, × 10^9^/L	NT	5.73	2.65	2.67	3.02	NA	2.76		3.09	2.6	3.4	NA	3.9
Eosinophils, × 10^9^/L	NT	5.44	29.94	9.18*	0.36	NA	0.23		17.85	7.4*	1.4	NA	0.5
Hemoglobin, g/dL	8.6	7.4	7.3	7.2	6.9	NA	7.7		12.4	12.6	12.4	NA	13.1
Aspartate aminotransferase (ref <50 UI/L)	NT	37	27	NA	NA	NA	26		43	NA	NA	NA	NA
Alanine aminotransferase (ref <60 UI/L)	NT	111	77	NA	NA	NA	27		68	NA	NA	NA	NA
Alkaline phosphatase (ref <130 UI/L)	NT	NA	150	NA	NA	NA	47		263	NA	NA	NA	NA
Gamma glutamyl transferase (ref <60 UI/L)	NT	183	229	NA	NA	NA	16		355	NA	NA	NA	NA
Antibody titers against *Acanthamoeba polyphaga mimivirus* virophage (microimmunofluorescence)													
IgG	Neg	100	200	NT	NT	100	50		400			100	50
IgM	Neg	100	100	NT	NT	0	0		100			0	0
IgA	Neg	0	25	NT	NT	0	0		0			0	0
Total antibody titers against mimivirus (microimmunofluorescence); significant threshold for total antibody titers ≥100	Neg	Neg	50	NT	NT	50	Neg		50			Neg	Neg
Molecular test results for *Acanthamoeba polyphaga mimivirus* virophage†													
Blood	NT	NT	Neg	NT	NT	Neg	Neg		Neg			Neg	Neg
Saliva	NT	NT	NT	NT	NT	Neg	Neg		NT			Neg	Neg
Feces	NT	NT	NT	Neg	NT	Neg	NT		NT	Neg		Neg	
Sputum	NT	NT	NT	NT	NT	NT	Neg		NT				Neg
Molecular test results for mimivirus‡													
Blood	NT	NT	Neg	NT	NT	Neg	Neg		Neg			Neg	Neg
Saliva	NT	NT	NT	NT	NT	Neg	Neg		NT			Neg	Neg
Feces	NT	NT	NT	Neg	NT	Neg	NT		NT	Neg		Neg	
Sputum	NT	NT	NT	NT	NT	NT	Neg		NT				Neg
Serologic test results													
Fascioliasis	NT	NT	NT	NT	NT	NT	NT		NT				
Hemagglutination	NA	1,280 (>160)	640	NT	Neg	NT	NT		320	160	160		
Western blot	NA	Pos	Neg	NT	NA	NT	NT		Neg		NA		
Toxocariasis, ELISA	NA	NA	1.06 (>0.41)	NT	1.01	NT	NT		0.54 (>0.48)		0.36		
Trichinellosis	NT	NT	NT	NT	NT	NT	NT		NT				
ELISA	NA	Pos	Pos	NT	Pos	NT	NT		Pos	Pos	Pos		
Western Blot	NA	NA	NT	NT	Neg	NT	NT		Pos		Neg		
Schistosomiasis	NA	Neg	Neg	NT	Neg	NT	NT		Neg		Neg		

*Patients returned from Laos on January 17, 2010. Treatment with praziquantel and albendazole started on February 2, 2010. Numbers in parentheses are cutoff values (i.e., values higher than cutoff values are positive). Ref, reference; APM, *Acanthamoeba polyphaga mimivirus;* NT, not tested; NA, not available; neg, negative; pos, positive. †APM virophage conventional PCR primers targeting the capsid-encoding gene included Spk_capsid_1F (5′-GTATATTGGACAGATCAAACTGGTG-3′) and Spk_capsid_30R (5′-CTCTCACCATAACCTACATT-3′). APM virophage real-time PCR primers and probe targeting the open reading frame–3 encoding gene included Sp3P (5′-TTTTTCCCTTTGAATTTCCCTCCAGCT-3′), Sp3F (5′-TGGAACAAACTTTCCTTCTGG-3′), and Sp3R (5′-AAAGATAGTAAACCGTTTGCAAAAAT-3′). ‡Giant virus real-time PCR primers and probe targeting heat shock protein 70–like protein encoding gene included MimiMama01 P (5′-CGGAATATTGCAAGTAACTGCACACGA-3′), MimiMama01 F (5′-ATCGGGATTTGAAAAAGGTC-3′), and MimiMama01 R (5′-CTTCCTTTTGCTCCCCATGT-3′).

We routinely test all serum samples for antibodies against intracellular microorganisms discovered at the World Health Organization Collaborative Center for Rickettsioses and Arthropod Borne Bacterial Diseases. For the patients reported here, we conducted microimmunofluorescence assays by using APM virophage and mamavirus APM strain antigens, obtained after amebal coculture with *Acanthamoeba castellanii*. From the first positive serum sample from each patient (acute-phase samples), we detected elevated IgG and IgM against the APM virophage (Table). For the woman, serum obtained when she had been pregnant, 5 months before disease onset, was negative. The APM antibody titer in the 3 samples was either negative or lower than the significant cutoff point (Table).

To determine specificity of the APM virophage antibodies, we tested 2 positive serum samples (1 from each patient) and the negative serum sample from the woman by Western blotting and 2-dimensional gel electrophoresis with purified APM virophage, mamavirus antigens, and *A. castellanii* antigens. Protein spots were excised from the silver-stained gels (Figure). All spots were excised for the APM virophage, and only immunoreactive spots were excised for mamavirus and *A. castellanii*. Peptide digestion and mass spectrum analyses were performed by using a matrix-assisted laser desorption ionization spectrometer (MALDI-TOF/TOF Bruker Ultraflex II; Bruker Daltonics, Wissembourg, France) (*2*). The negative serum showed no immune reaction against virophage proteins, whereas the positive serum showed high-intensity immune reactions for 2 groups of spots, identified as open reading frames (ORFs) 21 and 14 (Figure).

**Figure:**
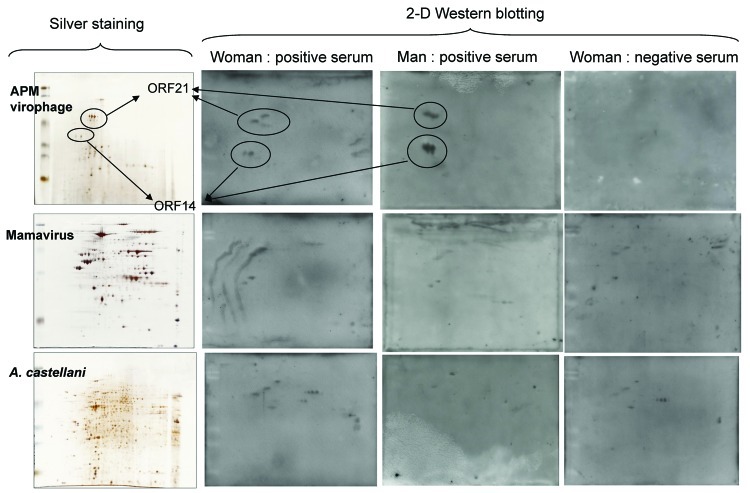
Two-dimensional (2-D) gel electrophoresis with silver stain results (on left) and Western blot results (on right) for 3 serum samples from patients who had visited Laos. The proteins were resolved by using 10% sodium dodecyl sulfate–polyacrylamide gel electrophoresis (Protean II xi chamber; Bio-Rad, Hercules, CA, USA). After migration, the gels were processed either by a silver-staining method compatible with mass spectrometry (*4*) or by transfer onto nitrocellulose membranes in a semidry blotting apparatus (Semi-Phor unit; Hoefer Scientific Instruments, San Francisco, CA, USA). The membranes were probed with horseradish peroxidase–conjugated goat anti-human secondary antibodies (Southern Biotech, Birmingham, AL, USA), and detection was achieved by enhanced chemiluminescence (ECL; GE Healthcare, Vélizy, France). APM, *Acanthamoeba polyphaga mimivirus*; ORF, open reading frame; *A. castellani*, *Acanthamoeba castellani*.

After protein spots were excised, we obtained a reference proteome map for APM virophages with only 2 identified ORFs (21 and 14), although several isoforms of these proteins were recovered from different parts of the gel (Figure). For the 2 patients, the immunoreactive spots corresponded to specific antibodies against virophage proteins. For mamavirus and *A. castellanii*, we observed immunoreactive spots of only slight intensity that were strictly identical in the 3 samples. Mass spectrometry was performed for these spots, but their identity was not verified. Whether these spots were artifacts or represented low antibody titers remains unknown. Various body fluids recovered during patient follow-up were screened with molecular testing (PCR and reverse transcription PCR with in-house primers and probes) and amebal coculture to seek APM virophages and/or APM. Results of molecular testing (Table) and amebal coculture (*2*,*5*) were negative.

Human exposure to virophages is unknown. We searched for environmental occurrences of APM virophage–like sequences in environmental metagenomic datasets (*6*). A BLASTP (http://blast.ncbi.nlm.nih.gov/Blast.cgi?PAGE=Protens) search of APM virophage-translated ORFs was performed against all metagenomic ORF peptides from Sanger reads (5,634,288,892 nt). These included translated peptide sequences of ORFs identified on all metagenomic sequence reads and excluded reads generated from Roche 454 GS-20 FLX and Titanium pyrosequencer (Roche Applied Science, Mannheim, Germany).

With no minimal e-value and an alignment of 25 reads per query, we obtained 347 hits from the different metagenomic datasets. When an increased stringency (e-value<10^−4^) was used, 112 reads were still recovered. Most hits (29 reads and 7 ORFs) were returned from the environment of Lake Gatun, a large artificial freshwater lake in the Republic of Panama. Numerous mimivirus-related sequences were also found in the Lake Gatun metagenome (561 reads with an e-value of <10^−4^ among 228 different ORFs), suggesting that the virophage and its host are common in this environment.

## Conclusions

Each patient was probably infected with a yet-unidentified parasite, although they each had positive test results for >1 foodborne helminthiases endemic to Southeast Asia. Nonetheless, the broad-spectrum antiparasitic treatment was effective (*7*).

For each patient, antibodies against the APM virophage were elevated. We cannot rule out serologic cross-reaction between APM virophage proteins and proteins of other origin, as described for the major capsid protein of *A. polyphaga* *mimivirus* that was recognized in serum of patients infected by *Francisella tularensis* (*8*). However, our method indicates that only human antibodies specific to APM virophage proteins were produced. The negative results of our molecular testing might have been caused by polymorphisms in the sequences chosen for amplification (*9*). Cross-reactivity with proteins of other virophages, such as Mavirus-infecting Cafeteria roenbergensis virus or Organic Lake virophage–infecting phycodnaviridae, is, however, the most plausible explanation because these virophages have some protein homology similar to that for capsid (*10*,*11*). Our environmental analysis indicated recovery of the APM virophage and viral host sequences from the environment, particularly a freshwater lake. Along with raw fish-borne parasites, aquatic environments could be a source of human exposure to the APM virophage, as they are for other ameba-associated microorganisms emerging as causal agents of pneumonia (*12*,*13*).

It is noteworthy that specific antibodies against the APM virophage but not APM were detected. Because the APM virophage is associated with a host giant virus, human exposure to APM virophages and to giant viruses should be concomitant. However, for the patients reported here, APM virophages might have been associated with an undescribed giant virus that cannot be detected with current laboratory techniques (*9*,*14*). Indeed, we have found that APM virophages infect distinct but related giant viruses of the family *Mimiviridae* (B. La Scola, unpub. data). Patients might have been directly exposed to free virophages that are apparently present in high numbers in fresh as well as saline water. For example, during the natural cycle involving virophages, phycodnaviruses, and algae, populations of each evolve over time (*10*). This potential direct exposure could explain why antibodies against giant viruses were not detected in the patients reported here. Virophages could also be associated with viruses that infect various protozoa or parasites (*15*).

We cannot exclude the possibility that each patient seroconverted while still in France, during the 5 months before their trip. It seems, however, more probable that they seroconverted while in Laos. Each patient ate raw fish, a potential source of the APM virophage. Human seroconversion against the APM virophage suggests that virophages could potentially be listed as emerging human pathogens.
